# Quantitative evaluation of gastrocnemius medialis mass in patients with chronic heart failure by gray-scale ultrasound and shear wave elastography

**DOI:** 10.3389/fcvm.2023.1132519

**Published:** 2023-03-09

**Authors:** Qiyu Yao, Yinglun Zhang, Jun Wu, Hua Shu, Xinhua Ye, Ao Li

**Affiliations:** ^1^ Department of Ultrasound, The First Affiliated Hospital of Nanjing Medical University, Nanjing, China; ^2^ Department of Geriatric Cardiology, The First Affiliated Hospital of Nanjing Medical University, Nanjing, China

**Keywords:** chronic heart failure, muscle mass, ultrasound, echo intensity, elastogarphy

## Abstract

**Objective:**

To assess the usefulness of gray-scale ultrasound (US) and shear wave elastography (SWE) in assessing the condition of the skeletal muscles in patients with chronic heart failure (CHF).

**Methods:**

We prospectively compared 20 patients with clinically diagnosed CHF and a control population of 20 normal volunteers. The gastrocnemius medialis (GM) of each individual in the rest and the contraction position was assessed using gray-scale US and SWE. The quantitative US parameters including the fascicle length (FL), pinnation angle (PA), echo intensity (EI), and Young's modulus of the muscle were measured.

**Results:**

In the CHF group compared with the control group, in the rest position, there was a significant difference in EI, PA, and FL of the GM (*P *< 0.001), but no statistically significant difference in Young's modulus values (*P *> 0.05); however, in the contraction position, all parameters were statistically different between the two groups (*P *< 0.001). In the different subgroups of the CHF group grouped according to New York Heart Association staging (NYHA) or left ventricular ejection fraction (LVEF), there were no significant differences in ultrasound parameters in the rest position. However, during the contraction of GM, the smaller the FL and Young's modulus, the larger the PA and EI with the increase of NYHA grade or the decrease of LVEF (*P *< 0.001).

**Conclusion:**

The gray-scale US and SWE can provide an objective assessment of skeletal muscle status for CHF patients and are expected to be used to guide their early rehabilitation training and improve their prognosis.

## 1. Introduction

Chronic heart failure (CHF) is a clinical syndrome characterized by compromised cardiac structure and function which leads to a decline in cardiac diastolic and systolic function as well as congestion of the systemic and pulmonary circulations. Due to the decrease in cardiac ejection function, CHF limits the energy and metabolism of body tissues, such as the loss of skeletal muscle mass and adipose tissue (1). The inflammatory reaction of skeletal muscle, the change of growth factor signal, the impairment of protein synthesis, and the enhancement of degradation (2) are the main mechanisms leading to the decrease of skeletal muscle mass in patients with CHF. What's worse, exercise intolerance in patients with heart failure can aggravate the decrease of muscle, which will further affect the quality of life and prognosis of patients with CHF. Exercise training is an important way in improving the quality of life and prognosis of patients with CHF. Some studies showed that it could improve skeletal muscle metabolism, growth factor activity and functional capacity, and attenuate muscle growth inhibitor expression and activation of the sympathetic and ubiquitin-proteasome systems (3). Greising et al. recommended early rehabilitation promotes the transformation of muscle fibers (4). Therefore, it is crucial to find an efficient method for early assessment of skeletal muscle status to guide early exercise rehabilitation in CHF patients.

Several methods have been applied in the evaluation of muscle quality. The biopsy is the gold standard for muscle quality evaluation, but it is invasive and traumatic. The dual energy x-ray can accurately evaluate the muscle mass by measuring the x-ray transmission in crossing tissues of the human body at two different energy levels, but it cannot effectively judge the degree of intramuscular steatosis (5). Computed tomography (CT) and magnetic resonance imaging (MRI) displayed great sensitivity, but the cost and contraindication make them impractical for regular muscle identification.

Ultrasound (US) has advantages over other imaging techniques, including non-radiation, portability, and real-time dynamics. The conventional gray-scale US can directly show the structure and shape of muscles and can assess muscle mass by measuring pinnation angle (PA) and muscle fascicle length (FL) (6). Echo intensity (EI) can be quantified by the computer-aided gray scale analysis software, which can reflect the changes in fibrous and adipose tissue in muscle (7). Previous studies indicated that EI was negatively correlated with muscle strength (8, 9). In addition, Shear wave elastography (SWE) is a new frontier of ultrasound, which can provide information regarding the tissue stiffness and has been used in the qualitative clinical diagnosis of breast and thyroid nodules (10, 11). The sensitivity and specificity of the SWE in assessing soft tissue were reported as good as magnetic resonance elastography (MRE) (12). Furthermore, SWE is expected to enable real-time quantitative assessment of the dynamic changes of muscle stiffness during dynamic stretching. The analogous studies have been performed on quantifying the status of the muscle in neuromuscular disorders like the Parkinson's disease or Duchenne muscular dystrophy (13, 14).

Therefore, this study was to investigate the viability of the gray-scale US combined with SWE for quantitative evaluation of muscle status in patients with CHF.

## 2. Methods

### 2.1. Study subjects

From February 2022 to July 2022, 24 patients with CHF from the Department of Cardiology of the first affiliated hospital of the Nanjing Medical University were collected. Meanwhile, 20 normal volunteers were recruited as controls, including 10 males and 10 females. The exclusion criteria were as follows: peripheral artery disease, lower limb joint disease, venous thrombosis, central nervous system disorder, and malignant tumors. All participants signed an informed consent form.

### 2.2. Demographic and clinical data

Demographic factors including age, sex and body mass index (BMI) were all considered.

The severity of CHF disease was mainly assessed by each patient's cardiac function based on the New York Heart Association staging (NYHA) and left ventricular ejection fraction (LVEF) according to the biplane Simpson estimate method. Therefore, in this study we divided the CHF group into three subgroups according to NYHA: I, II, and III-IV. Also, the CHF group was divided into three subgroups according to LVEF: (1) heart failure with reduced ejection fraction (HFrEF): LVEF <40%; (2) heart failure with midrange ejection fraction (HFmrEF): LVEF between 40% to 50%; (3) heart failure with preserved ejection fraction (HFpEF): LVEF>50%.

### 2.3. Study protocol

The ultrasound examinations were performed with a US system (Aixplorer; SuperSonic Imagine, Aix en Provence, France) equipped with a 4–15 MHz liner transducer by two sonographers who had more than 3 years of experience in musculoskeletal ultrasound. The gray-scale US parameters were set to a depth of 3.0 cm, a gain of 72 dB, and moderate time gain compensation, and remained unchanged throughout the study period.

**Figure 1:**
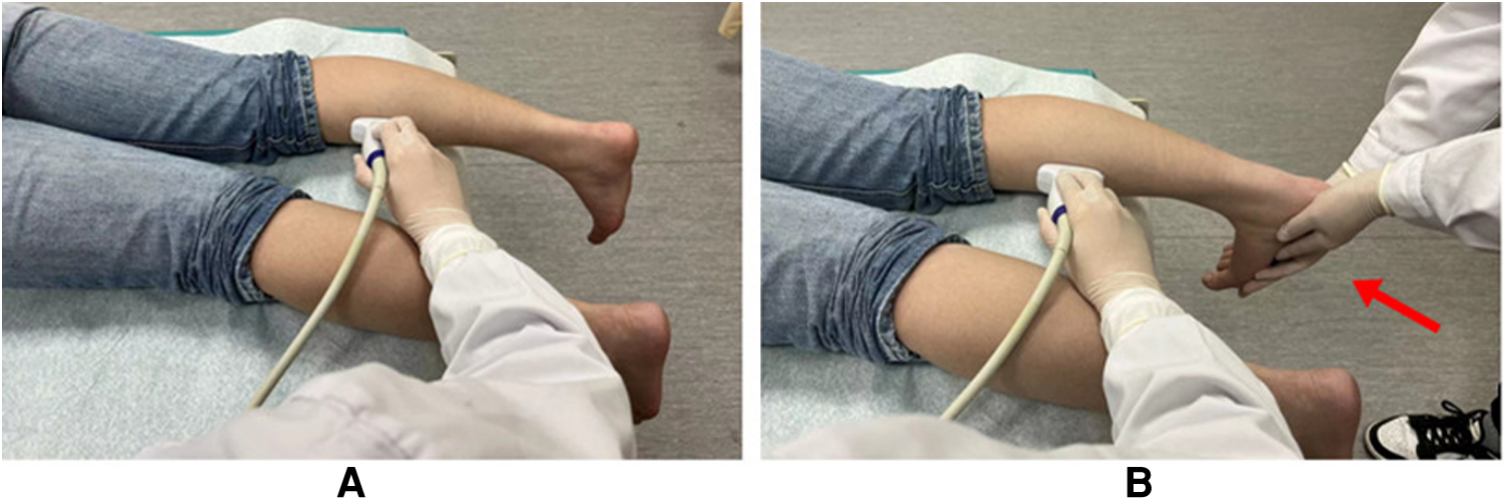
Photos of the rest position (**A**) and contraction position (**B**) under multimodal ultrasound examination. The extension of the foot's dorsum under the contraction position is indicated by the red arrow.

The gastrocnemius medialis (GM) muscle was selected as the target muscle for ultrasound scanning. Participants were informed to take a prone position and let their feet droop naturally. This position was defined as the rest position (Figure 1A). The probe was placed longitudinally at the thickest part of the muscle for 2 min, with conventional ultrasound images were retained every 20 s. After a 10-minute rest break, the probe was placed in the same position for the SWE examination. The image was captured every 20 s during the 2-minute examination. After that, the dorsum of the foot was fully stretched to the ventral side of the calf to achieve maximum tension with the assistance of another physician. This position was defined as the contraction position (Figure 1B). The experimental method for the contraction position was the same as that for the rest position.

### 2.4. Image analysis

**Figure 2:**
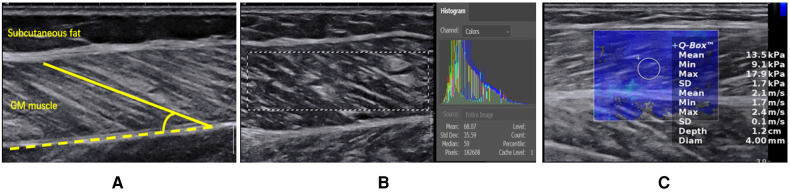
(**A**) Measurement of muscle fascicle length and PA by gray-scale ultrasound on the long axis of GM. The yellow dotted line indicates the deep aponeurosis of GM, and the yellow solid line indicates the muscle fascicle length. The angle between the muscle fascicle and the deep aponeurosis is PA. (**B**) EI analysis of GM muscle by using adobe Photoshop cc 2021 software. The mean gray value was shown in the histogram interface. (**C**) Measurement of the Young's modulus of GM.

(1) Muscle FL and PA in gray-scale ultrasound images: FL was measured as the length of the hyperechoic muscle fascicle between the superficial and deep aponeurosis. PA was measured as the angle between muscle fascicles and deep aponeurosis (15) (Figure 2A). (2) EI: The average gray value was analyzed through the histogram interface using adobe Photoshop cc 2021 software. The region of interest (ROI) measured in GM muscle did not include the aponeurosis (Figure 2B). (3) Young's modulus in SWE: Q-box with diameter of 4 mm was used for measurement, and the average Young's modulus was recorded. The final result was the average of three measurements at different positions in the same image (Figure 2C).

### 2.5. Statistical analysis

The data were analyzed using SPSS 26.0 statistical analysis software. Count data were tested for normality using the *χ*^2^ test, and measurements conforming to a normal distribution were expressed as mean ± SD using the Kolmogorov-Smirnov test. If the distribution was normal, two groups of measurements were analyzed using the independent sample *t*-test, and multiple groups of measurements were compared utilizing single factor analysis of variance. If the distribution was skewed, the Scheirer-Ray-Hare test, a two-factor ANOVA non-parametric test, was used for comparisons. *P* < 0.05 was considered a statistically difference.

## 3. Results

### 3.1. Demographics

Of the 24 patients with CHF, two participants who complained of muscle laceration and venous thrombosis during the informed consent discussion and two participants who were unable to sustain 2 min of dorsiflexion in contraction position were excluded from the study. Finally, a total of 20 patients with CHF (10 males and 10 females, mean age ± SD: 58.85 ± 2.51 years) and 20 healthy volunteers (9 males and 11 females, mean age ± SD: 58.00 ± 3.35 years) were included in this study. The general data of the subjects in both groups are shown in (Table 1). There were no statistically significant differences between the CHF and control groups in terms of age (*P* = 0.45), gender (*P* = 0.87) and BMI (*P* = 0.25).

**Table 1:** Demographic parameters of the CHF group and the control group.

Characteristic	Age (years)	Sex (male/female)	BMI (kg/m^2^)
CHF group	58.85 ± 2.51	10/10	22.53 ± 0.59
Control group	58.00 ± 3.35	9/11	22.20 ± 0.77
*t* value	0.96		1.51
*P* value	0.45	0.75	0.25

### 3.2. Comparison of the ultrasound parameters in the rest position between the CHF group and the control group

Table 2 showed significant differences in the FL (*P* < 0.001) and PA (*P* < 0.001) between the CHF group and the control group. Moreover, The EI was significantly higher in the CHF group than in the control group (*P* < 0.001). However, no difference was found in Young's modulus of the GM muscle (*P* = 0.66).

**Table 2:** Comparison of the ultrasound parameters in the rest position between the CHF group and the control group.

	Participants	Muscle fascicle length (mm)	Pinnation angle (degree)	Echo intensity	Young's modulus (Kpa)
CHF group	20	31.50 ± 0.27	17.60 ± 1.39	78.76 ± 5.00	8.81 ± 0.48
Control group	20	39.42 ± 0.39	21.29 ± 2.33	60.08 ± 5.21	8.36 ± 0.84
*t* value		71.50	5.94	11.51	1.92
*P* value		<0.001	<0.001	<0.001	0.66

### 3.3. Comparison of the ultrasound parameters in the contraction position between the CHF group and the control group

**Figure 3:**
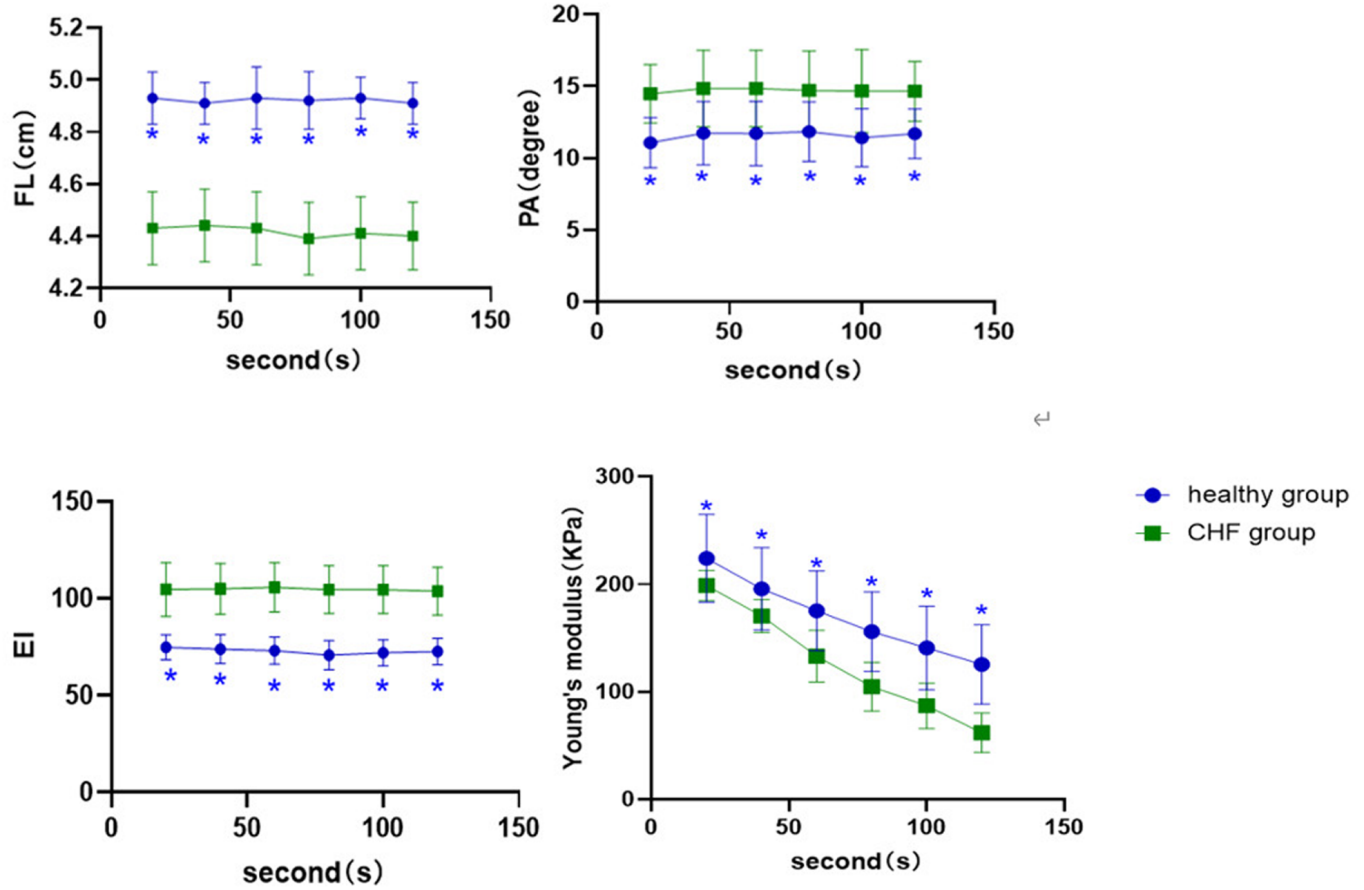
Comparison of ultrasound parameters at different time points of the contraction position between control group and CHF group. *: compared with CHF group; the difference is statistically significant.

At each time point of the contraction position, (Figure 3) showed that the FL (*P* < 0.001) and EI of the control group (*P* < 0.001) were higher than those of the CHF group, whereas PA was lower than that of the CHF group (*P* < 0.05). In the SWE examination, Young's modulus decreased with increasing contraction time in both groups. Meanwhile, Young's modulus of the control group was significantly higher than that of the CHF group at each time point (*P* < 0.05).

### 3.4. Comparison of the ultrasound parameters among different subgroups of CHF patients

In the rest position, no differences were found in FL(*P* = 0.90), PA(*P* = 0.41), EI (*P* = 0.12) and Young's modulus (*P* = 0.21) among the NYHA subgroups (Table 3). Similarly, all ultrasound parameters showed no significant differences in the three groups of the LVEF grading (*P* > 0.05, respectively; Table 4).

**Table 3:** The ultrasound parameters of GM in rest position among the NYHA subgroups of the CHF group.

	Participants	Muscle fascicle length (mm)	Pinnation angle (degree)	Echo intensity	Young's modulus (Kpa)
NYHA I	6	33.98 ± 1.05	17.83 ± 2.40	77.40 ± 2.30	8.48 ± 0.23
NYHA II	8	34.18 ± 0.84	18.37 ± 1.18	81.54 ± 4.77	8.82 ± 0.55
NYHA III–IV	6	33.93 ± 1.46	19.33 ± 2.16	76.41 ± 6.05	8.93 ± 0.43
*F* value		0.10	0.95	2.45	1.70
*P* value		0.90	0.41	0.12	0.21

**Table 4:** The ultrasound parameters of GM in rest position among the LVEF subgroups of the CHF group.

	Participants	Muscle fascicle length (mm)	Pinnation angle (degree)	Echo intensity	Young's modulus (Kpa)
HFpEF	6	34.07 ± 1.09	17.33 ± 1.96	79.71 ± 4.16	8.75 ± 0.41
HFmrEF	7	34.14 ± 0.62	19.57 ± 1.61	79.67 ± 5.07	8.41 ± 0.89
HFrEF	7	33.94 ± 1.48	18.42 ± 1.72	77.03 ± 5.80	8.14 ± 1.11
*F* value		0.57	2.61	0.62	0.77
*P* value		0.94	0.10	0.55	0.48

**Figure 4:**
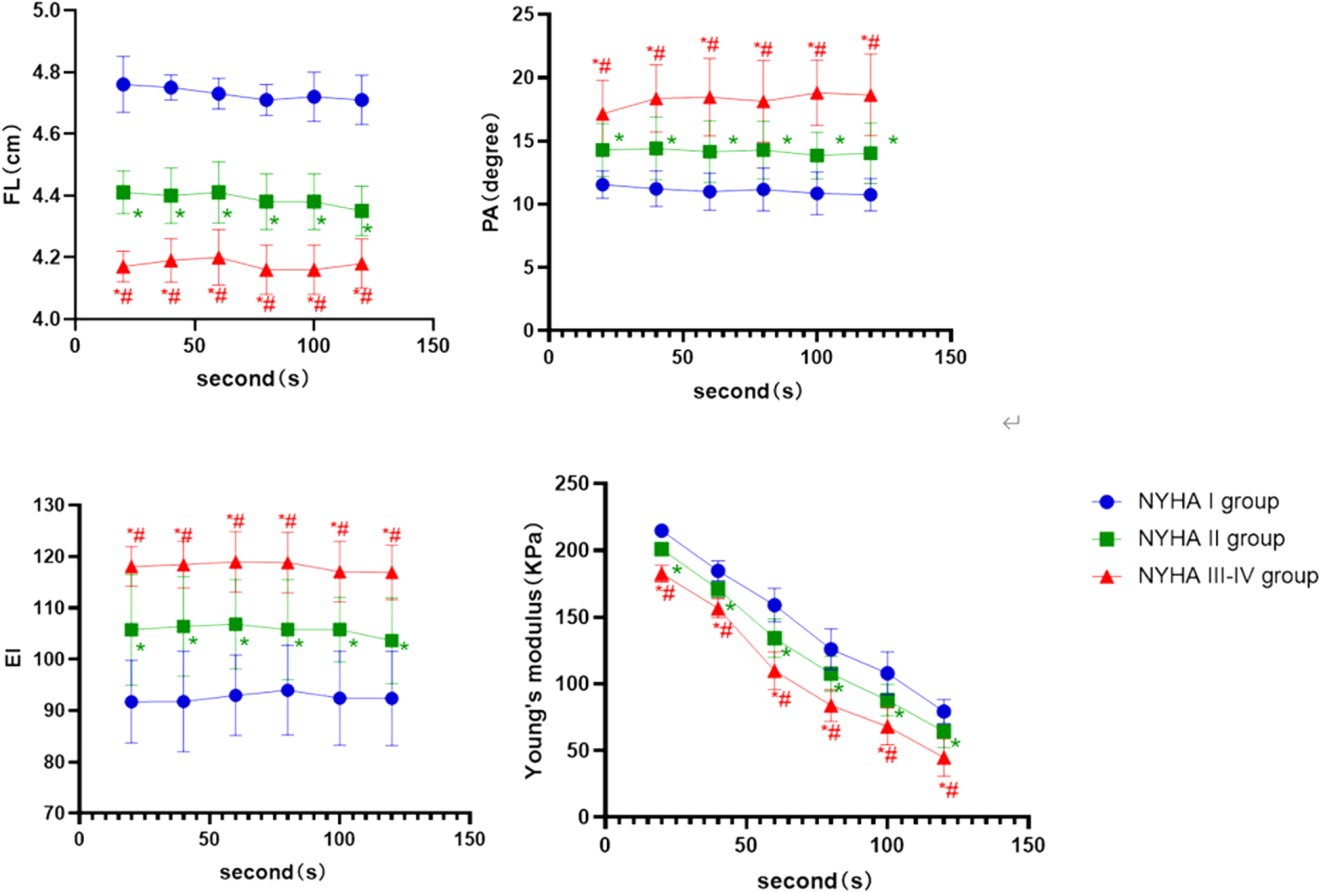
The boxplot graphic showed the changes in the ultrasound parameters over time for each NYHA subgroup of CHF group in the contraction position. *P* < 0.05 was considered a statistically difference. *: compared with NYHA I group; the difference is statistically significant. #: compared with NYHA II, the difference is statistically significant.

In the contraction position, (Figure 4) showed that the FL and Young's modulus were higher in the NYHA I group than in the NYHA II and III-IV group at each time point (*P* < 0.001, *P* < 0.05, respectively), whereas they were higher in the NYHA II group than in the NYHA III–IV group (*P* < 0.001). EI and PA increased with increasing NYHA grade, and the difference among the three groups was statistically significant (*P* < 0.001, *P* < 0.05, respectively).

**Figure 5:**
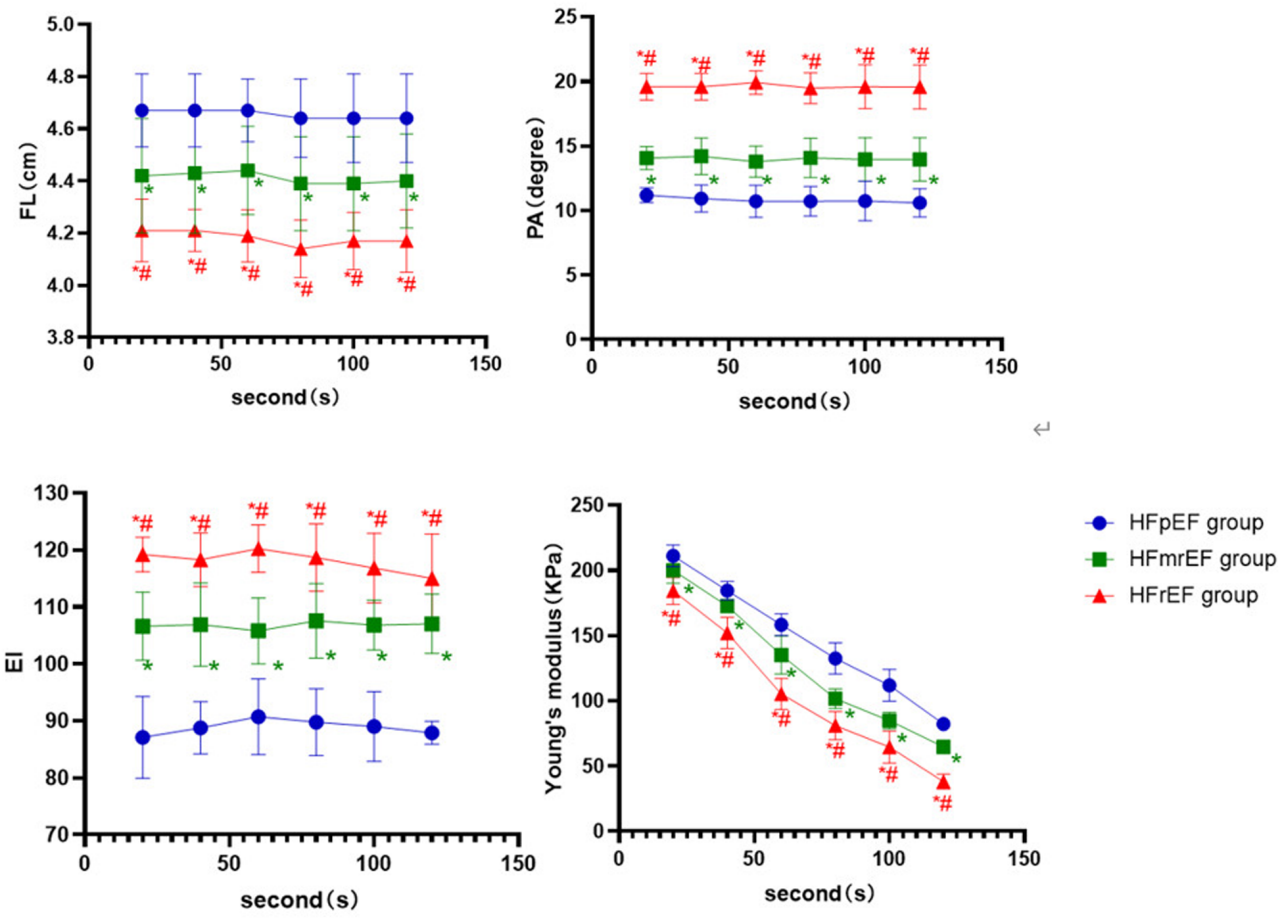
The boxplot graphic showed the changes in the ultrasound parameters over time for each LVEF subgroup of CHF group in the contraction position. *P* < 0.05 was considered a statistical difference. *: compared with HFpEF group, the difference is statistically significant. #: compared with HFmrEF, the difference is statistically significant.

Among the three subgroups of the LVEF classification in the contraction position, the HFpEF group had longer FL and higher Young's modulus than the HFmrEF and HFrEF group (*P* < 0.001, *P* < 0.05, respectively). The PA and EI in the HFpEF group were lower than in the other two groups (*P* < 0.001), while the HFmrEF group was lower than the HFrEF group. The differences indicated above were statistically significant (*P* < 0.05, respectively, Figure 5).

## 4. Discussion

The typical clinical symptoms and signs of CHF include fatigue, increased jugular venous pressure and dyspnea. CHF patients are often accompanied by physical intolerance, such as weight loss and muscle atrophy. Low skeletal muscle mass is now an independent prognostic factor for patients with CHF (16). Early rehabilitation training can improve the quality of life and also reduce mortality and hospitalization rates in patients with mild-to-moderate CHF (17). Therefore, it is necessary to evaluate muscle quality quantitatively at an early stage. However, the dynamic tissue biomechanical properties of skeletal muscle make it impossible to analyze it in detail by a single imaging frame, or in a static or constant position. In comparison to CT, MR, and DXA, US has good reproducibility and allows for rapid, dynamic analysis of muscle. The purpose of this study was to explore the application of the US in detecting peripheral muscle status in CHF patients.

In this study, we chose the GM muscle as the target muscle because it is the dominant muscle in the triceps calf complex during passive dorsiflexion. Its shallow location and large volume make it easy for doctors to localize and measure ultrasound parameters (18).

FL and PA are the parameters that can represent the biomechanical characteristics of the muscles. Muscle strength when the muscular was at rest was positively associated with PA and FL (6). With an increase in muscle contractile force, the PA decreased, and the FL became longer (19, 20). The comparison between the control group and the CHF group showed that FL and PA of the GM were higher in the rest position. In the contraction position, FL was longer in the control group than in the CHF group, while the PA was smaller. The results showed that in both the resting and contraction positions, the muscle mass of the CHF group was less than that of the healthy group. The primary reason for these differences might be structural changes in the skeletal muscle in CHF patients, such as muscle atrophy and muscle fiber transformation (21, 22).

This study also found that the EI of the CHF group was significantly higher than that of the control group, whether in rest or contraction position. Previous studies have shown that the increase in EI indicated an increase in the degree of fat infiltration in muscle (23). The increased EI in the CHF group also indicated intramuscular fat infiltration. Fat-infiltrated skeletal muscles produce inflammatory cytokines which can enhance insulin resistance, muscle catabolism, and mitochondrial dysfunction (24). It may cause a direct catabolic effect on skeletal muscle and impair skeletal muscle protein synthesis, leading to a loss of muscle mass (24, 25). Previous studies showed that the EI of muscle was negatively correlated with muscle mass. EI could initially assess the level of muscle fat infiltration and reflect muscle quality to direct therapeutic rehabilitation (26, 27).

The SWE examination showed no difference between the two groups in the rest position. It inferred that the skeletal muscle of CHF patients was almost unaffected in the rest position. In contrast, the Young's modulus value in the contraction position of CHF group was significantly lower than that of the control group, which meant that the force generated by the GM muscle passive extension process of CHF patients due to anatomical or functional changes was less than that of healthy people. Maslarska surmised that the exercise intolerance of the CHF patients manifested as reduced muscle stiffness and muscle mass in the SWE examination (28), and our results agreed with these findings.

Furthermore, CHF patients were classified according to NYHA and LVEF in this study. There were no significant differences in ultrasound parameters between the NYHA and LVEF subgroups in the rest position. In the contraction position, PA and Young's modulus decreased in each subgroup while the FL and EI increased. It indicated that muscle mass decreased as NYHA levels increased and LVEF decreased. Kitzman's studies found that, compared to the other two groups, type I muscle fibers were more easily transformed into type II in HFrEF (29). The HFrEF patients were also accompanied by worsening of muscle fiber atrophy and abnormal mitochondrial function, leading to a decline in exercise capacity and muscle mass. NYHA was graded by clinical symptoms, demonstrating that as the grade increased and symptoms worsen, the muscle hardness reduced and activity restriction grew more severe. Thus, early intervention and treatment are needed (30, 31).

There are some limitations in current study. First, this study only included one single center and had a small sample size. Further confirmation with multi-center and a larger sample size for follow-up is required. Second, it was a cross-sectional study. More studies are needed to determine whether the disease duration or following exercise rehabilitation can have effect on changes in ultrasound parameters. Additionally, not all participants had muscle biopsies. Therefore, we could not identify the precise cause and origin of the muscle abnormalities.

In conclusion, the US technology including gray-scale US and SWE could provide quantitative parameters such as FL, PA, EI, and Young's modulus value to evaluate the quality of the GM muscle in different status. It is expected to become a reliable technique for assessing skeletal muscle abnormalities in CHF patients, and provide an objective reference for guiding early rehabilitation training and improving the prognosis of CHF patients.

## Data Availability

The raw data supporting the conclusions of this article will be made available by the authors, without undue reservation.
