# Supplementary material for: Quantitative evaluation of gastrocnemius medialis mass in patients with chronic heart failure by gray-scale ultrasound and shear wave elastography
Source: Front Cardiovasc Med. 2023 Mar 9;10:1132519. doi: 10.3389/fcvm.2023.1132519 (PMC10034337; doi:10.3389/fcvm.2023.1132519)
Supplement: Supplementary file 1 [file Table1.docx]

Supplementary Material

Quantitative Evaluation of Gastrocnemius Medialis Mass in Patients with Chronic Heart Failure by gray-scale ultrasound and shear wave elastography

Qiyu Yao^1 †^, Yinglun Zhang^1 †^, Jun Wu^2^, Hua Shu^1^, Xinhua Ye^1^ ^*^, Ao Li^1^ ^*^

1. Department of Ultrasound, the First Affiliated Hospital of Nanjing Medical University, Nanjing, China
2. Department of Geriatric Cardiology, the First Affiliated Hospital of Nanjing Medical University, Nanjing, China

***First Authors:***

†Qiyu Yao and Yinglun Zhang have contributed equally to this work and share first authorship.

***Corresponding authors:***

These authors have contributed equally to this work and share Corresponding authors

Xinhua Ye **^*^**:

Department of Ultrasound, The First Affiliated Hospital of Nanjing Medical University, 300 Guangzhou Road, Nanjing 210029, China.

E-mail: yexh-0125@163.com

Ao Li **^*^**:

Department of Ultrasound, The First Affiliated Hospital of Nanjing Medical University, 300 Guangzhou Road, Nanjing 210029, China.

E-mail: cqh2liao@163.com.

Table 1: Comparison of the muscle fascicle length(mm) of GM in the contraction position between the CHF group and the control group.

|  | Participants | 20s | 40s | 60s | 80s | 100s | 120s |
| --- | --- | --- | --- | --- | --- | --- | --- |
| CHF group | 20 | 44.3±2.48 | 44.4±0.24 | 44.3±2.33 | 43.9±1.10 | 44.1±1.43 | 44.0±2.39 |
| Control group | 20 | 49.3±1.01 | 49.1±0.85 | 49.3±1.21 | 49.3±0.83 | 49.3±0.84 | 49.2±0.87 |
| t value |  | 7.69 | 7.74 | 8.04 | 8.28 | 8.43 | 8.36 |
| P value |  | <0.001 | <0.001 | <0.001 | <0.001 | <0.001 | <0.001 |

Table 2: Comparison of the PA (degree) of GM in the contraction position between the CHF group and the control group.

|  | Participants | 20s | 40s | 60s | 80s | 100s | 120s |
| --- | --- | --- | --- | --- | --- | --- | --- |
| CHF group | 20 | 14.47±2.04 | 14.83±2.66 | 14.83±2.66 | 14.70±2.74 | 14.67±2.89 | 14.65±2.09 |
| Control group | 20 | 11.06±1.75 | 11.72±2.21 | 11.71±2.24 | 11.83±2.07 | 11.41±2.03 | 11.68±1.73 |
| t value |  | 4.07 | 3.05 | 2.81 | 2.80 | 3.12 | 2.78 |
| P value |  | <0.001 | 0.004 | 0.008 | 0.008 | 0.004 | 0.009 |

Table 3: Comparison of the EI of GM in the contraction position between the CHF group and the control group.

|  | Participants | 20s | 40s | 60s | 80s | 100s | 120s |
| --- | --- | --- | --- | --- | --- | --- | --- |
| CHF group | 20 | 104.54±13.96 | 104.87±13.09 | 105.62±12.88 | 104.48±12.34 | 104.48±12.34 | 103.69±12.28 |
| Control group | 20 | 74.68±6.46 | 73.73±7.57 | 73.01±7.04 | 70.61±7.53 | 71.92±6.68 | 72.50±6.82 |
| t value |  | 8.10 | 8.65 | 9.31 | 9.77 | 9.72 | 9.30 |
| P value |  | <0.001 | <0.001 | <0.001 | <0.001 | <0.001 | <0.001 |

Table 4: Comparison of the Young’s modulus (Kpa) of GM in the contraction position between the CHF group and the control group.

|  | Participants | 20s | 40s | 60s | 80s | 100s | 120s |
| --- | --- | --- | --- | --- | --- | --- | --- |
| CHF group | 20 | 198.63±14.06 | 170.30±15.34 | 133.06±24.11 | 104.71±22.64 | 86.85±21.11 | 61.91±18.31 |
| Control group | 20 | 223.85±40.90 | 195.68±38.41 | 175.12±37.18 | 155.82±36.80 | 140.62±38.82 | 125.20±36.93 |
| t value |  | 2.43 | 2.74 | 4.14 | 5.17 | 5.34 | 6.75 |
| P value |  | 0.02 | 0.009 | <0.001 | <0.001 | <0.001 | <0.001 |

Table 5: The muscle fascicle length (mm) of GM in contraction position among the NYHA subgroups of the CHF group.

|  | Participants | 20s | 40s | 60s | 80s | 100s | 120s |
| --- | --- | --- | --- | --- | --- | --- | --- |
| NYHA I | 6 | 47.63±0.29 | 47.53±0.43 | 47.30±0.56 | 47.10±0.55 | 47.18±0.85 | 47.18±0.85 |
| NYHA II | 8 | 44.13±0.77 | 44.09±0.91 | 44.11±1.06 | 43.79±0.76 | 43.80±0.92 | 43.52±0.76 |
| NYHA III-IV | 6 | 41.79±0.55 | 41.94±0.75 | 42.04±0.92 | 41.37±0.93 | 41.64±0.81 | 41.81±0.86 |
| F value |  | 161.74 | 94.31 | 56.89 | 87.33 | 67.15 | 61.72 |
| P value |  | <0.001 | <0.001 | <0.001 | <0.001 | <0.001 | <0.001 |

Table 6: The PA (degree) of GM in contraction position among the NYHA subgroups of the CHF group.

|  | Participants | | 20s | 40s | 60s | 80s | 100s | 120s |
| --- | --- | --- | --- | --- | --- | --- | --- | --- |
| NYHA I | | 6 | 11.54±1.08 | 11.22±1.41 | 10.99±1.47 | 11.17±1.71 | 10.85±1.71 | 10.75±1.29 |
| NYHA II | | 8 | 14.29±2.11 | 14.41±2.49 | 14.14±2.45 | 14.28±2.27 | 13.85±1.83 | 14.02±2.39 |
| NYHA III-IV | | 6 | 17.15±2.62 | 18.35±2.65 | 18.46±3.06 | 18.13±3.22 | 18.80±2.59 | 18.63±3.22 |
| F value | |  | 11.67 | 15.78 | 15.08 | 12.41 | 24.04 | 15.29 |
| P value | |  | 0.001 | <0.001 | <0.001 | <0.001 | <0.001 | <0.001 |

Table 7: The EI of GM in contraction position among the NYHA subgroups of the CHF group.

|  | Participants | 20s | 40s | 60s | 80s | 100s | 120s |
| --- | --- | --- | --- | --- | --- | --- | --- |
| NYHA I | 6 | 91.72±8.01 | 91.75±9.80 | 92.98±7.84 | 93.98±8.80 | 92.42±9.18 | 92.41±9.18 |
| NYHA II | 8 | 105.74±10.78 | 106.40±9.64 | 106.82±8.69 | 105.78±9.74 | 105.78±6.27 | 103.63±8.39 |
| NYHA III-IV | 6 | 118.10±3.90 | 118.41±4.53 | 118.98±5.91 | 118.85±5.91 | 117.06±5.93 | 116.92±5.34 |
| F value |  | 13.35 | 21.01 | 19.50 | 14.04 | 18.31 | 17.76 |
| P value |  | <0.001 | <0.001 | <0.001 | <0.001 | <0.001 | <0.001 |

Table 8: The Young’s modulus (Kpa) of GM in contraction position among the NYHA subgroups of the CHF group.

|  | Participants | 20s | 40s | 60s | 80s | 100s | 120s |
| --- | --- | --- | --- | --- | --- | --- | --- |
| NYHA I | 6 | 214.83±3.26 | 184.65±7.55 | 158.95±12.51 | 125.85±15.26 | 107.83±15.89 | 79.28±9.01 |
| NYHA II | 8 | 200.86±2.76 | 170.95±6.08 | 134.18±14.41 | 107.51±13.15 | 87.70±11.89 | 64.20±11.96 |
| NYHA III-IV | 6 | 182.51±6.18 | 156.57±7.31 | 109.74±14.01 | 83.80±11.92 | 68.01±13.81 | 44.74±14.11 |
| F value |  | 88.74 | 11.35 | 20.76 | 12.49 | 12.75 | 13.51 |
| P value |  | <0.001 | 0.001 | <0.001 | <0.001 | <0.001 | <0.001 |

Table 9: The muscle fascicle length (mm) of GM in contraction position among the LVEF subgroups of the CHF group.

|  | Participants | 20s | 40s | 60s | 80s | 100s | 120s |
| --- | --- | --- | --- | --- | --- | --- | --- |
| HFpEF | 6 | 46.73±1.49 | 46.61±1.45 | 46.71±1.23 | 46.34±1.50 | 46.40±1.71 | 46.40±1.89 |
| HFmrEF | 7 | 44.27±2.20 | 44.35±2.30 | 44.34±1.79 | 43.92±1.88 | 44.06±1.89 | 43.95±1.73 |
| HFrEF | 7 | 42.10±1.20 | 42.15±0.84 | 41.98±1.07 | 41.45±1.09 | 41.71±1.19 | 41.75±1.01 |
| F value |  | 10.47 | 9.95 | 15.84 | 15.04 | 11.97 | 12.54 |
| P value |  | 0.001 | 0.001 | <0.001 | <0.001 | 0.001 | <0.001 |

Table 10: The PA (degree) of GM in contraction position among the LVEF subgroups of the CHF group.

|  | Participants | 20s | 40s | 60s | 80s | 100s | 120s |
| --- | --- | --- | --- | --- | --- | --- | --- |
| HFpEF | 6 | 11.18±0.58 | 10.93±1.05 | 10.71±1.23 | 10.72±1.15 | 10.74±1.53 | 10.59±1.10 |
| HFmrEF | 7 | 14.06±0.89 | 14.19±1.43 | 13.79±1.20 | 14.08±1.53 | 13.95±1.68 | 13.95±1.68 |
| HFrEF | 7 | 19.59±1.02 | 19.59±1.03 | 19.92±0.90 | 19.49±1.21 | 19.58±1.70 | 19.57±1.71 |
| F value |  | 60.57 | 77.21 | 103.42 | 65.85 | 44.36 | 100.67 |
| P value |  | <0.001 | <0.001 | <0.001 | <0.001 | <0.001 | <0.001 |

Table 11: The EI of GM in contraction position among the LVEF subgroups of the CHF group.

|  | Participants | 20s | 40s | 60s | 80s | 100s | 120s |
| --- | --- | --- | --- | --- | --- | --- | --- |
| HFpEF | 6 | 87.11±7.20 | 88.78±4.58 | 90.73±6.67 | 89.78±5.86 | 89.03±6.10 | 87.92±2.00 |
| HFmrEF | 7 | 106.62±6.00 | 106.90±7.32 | 105.84±5.78 | 107.56±6.53 | 106.82±4.37 | 107.01±5.21 |
| HFrEF | 7 | 119.20±3.04 | 118.28±4.73 | 120.23±4.18 | 118.69±5.94 | 116.82±6.13 | 115.03±7.84 |
| F value |  | 47.88 | 38.21 | 40.79 | 33.59 | 39.95 | 38.57 |
| P value |  | <0.001 | <0.001 | <0.001 | <0.001 | <0.001 | <0.001 |

Table 12: The Young’s modulus (Kpa) of GM in contraction position among the LVEF subgroups of the CHF group.

|  | Participants | 20s | 40s | 60s | 80s | 100s | 120s |
| --- | --- | --- | --- | --- | --- | --- | --- |
| HFpEF | 6 | 211.08±8.32 | 184.45±7.08 | 158.20±8.41 | 132.41±12.00 | 111.85±12.17 | 82.18±4.7700 |
| HFmrEF | 7 | 200.07±9.90 | 172.67±5.13 | 135.08±14.60 | 101.68±7.60 | 84.85±6.50 | 64.65±4.331 |
| HFrEF | 7 | 184.25±10.34 | 152.08±12.09 | 105.21±12.00 | 81.05±10.75 | 64.53±12.24 | 38.00±5.81 |
| F value |  | 11.86 | 22.23 | 28.06 | 40.10 | 32.22 | 121.99 |
| P value |  | 0.001 | <0.001 | <0.001 | <0.001 | <0.001 | <0.001 |
